# Supplementary material for: The Potential of Food Fortification as an Enabler of More Environmentally Sustainable, Nutritionally Adequate Diets
Source: Nutrients. 2023 May 25;15(11):2473. doi: 10.3390/nu15112473 (PMC10255058; doi:10.3390/nu15112473)
Supplement: Supplementary file 1 [file nutrients-15-02473-s001.zip › Supplementary Figure S1.pdf]

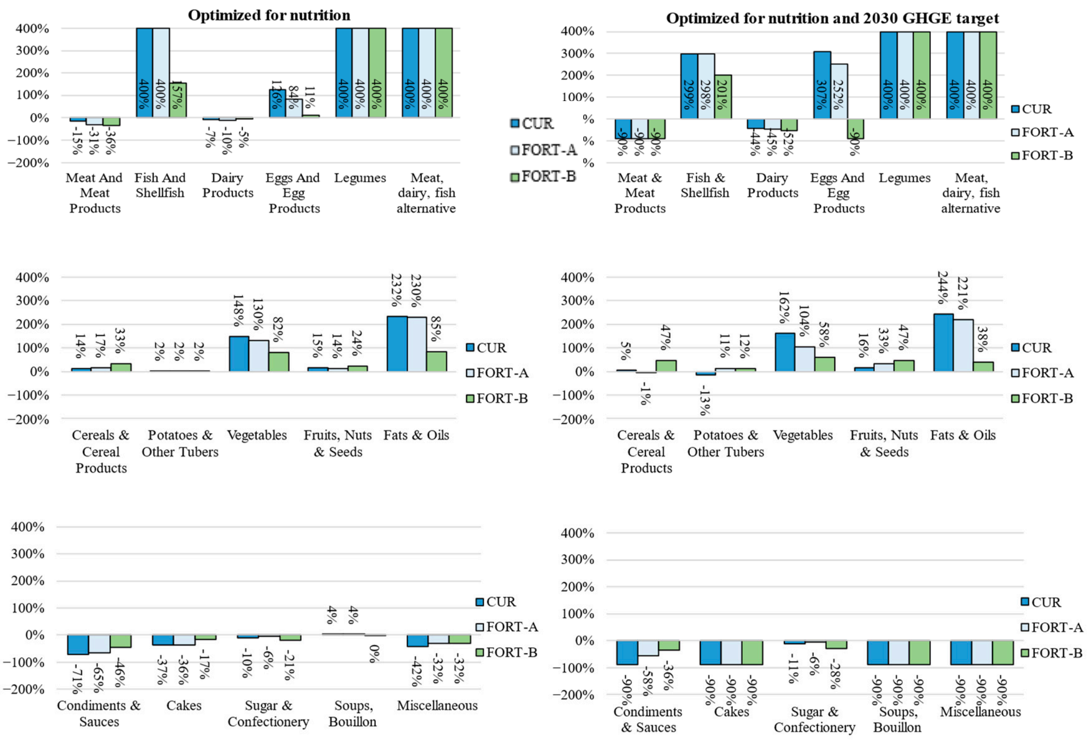
Optimized for nutrition
Optimized for nutrition and 2030 GHGE target
Meat And Meat Products
Fish And Shellfish
Dairy Products
Eggs And Egg Products
Legumes
Meat, dairy, fish alternative
Meat & Meat Products
Fish & Shellfish
Cereals & Cereal Products
Potatoes & Other Tubers
Vegetables
Fruits, Nuts & Seeds
Fats & Oils
Condiments & Sauces
Cakes
Sugar & Confectionery
Soups, Bouillon
Miscellaneous
CUR
FORT-A
FORT-B

(b) Women

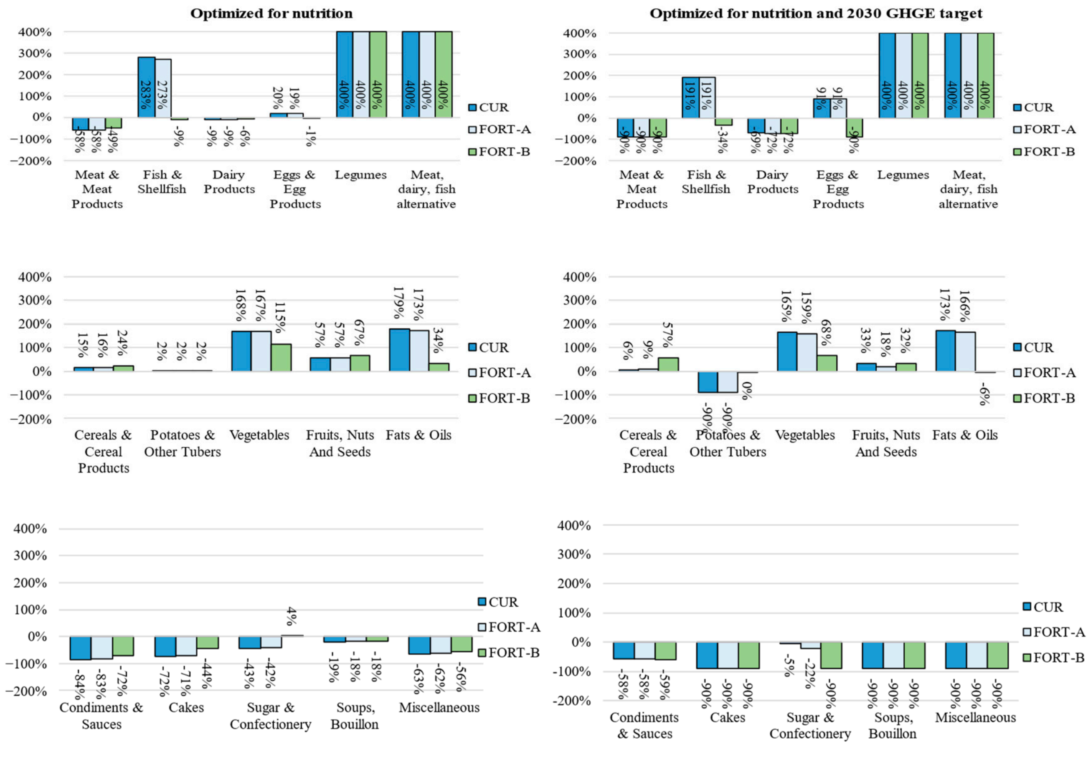
Optimized for nutrition
Optimized for nutrition and 2030 GHGE target
Meat & Meat Products
Fish & Shellfish
Dairy Products
Eggs & Egg Products
Legumes
Meat, dairy, fish alternative
Cereals & Cereal Products
Potatoes & Other Tubers
Vegetables
Fruits, Nuts And Seeds
Fats & Oils
Condiments & Sauces
Cakes
Sugar & Confectionery
Soups, Bouillon
Miscellaneous
CUR
FORT-A
FORT-B

(**b**) Men

Supplementary Figure S1. Percent change in consumed quantity (g/d) of food groups, relative to baseline diet among Dutch women (**a**) and men (**b**) aged 19–30 years, for three fortification scenarios: current diet (CUR); CUR with fortified meat, dairy, and fish alternatives (FORT-A); FORT-A with fortified bread and oils (FORT-B), optimized for nutritional adequacy and 2030 greenhouse gas emission (GHGE) targets within acceptable boundaries on food groups of 10–500% of current intake.
